# Listening as a path to psychological discovery: an introduction to the Listening Guide

**DOI:** 10.1007/s40037-017-0335-3

**Published:** 2017-03-27

**Authors:** Carol Gilligan, Jessica Eddy

**Affiliations:** 1 New York University, New York City, NY USA; 2 Derner Institute of Advanced Psychological Studies, Adelphi University, New York, USA

*A Qualitative Space* highlights research approaches that push readers and scholars deeper into qualitative methods and methodologies. Contributors to *A Qualitative Space *may: advance new ideas about qualitative methodologies, methods, and/or techniques; debate current and historical trends in qualitative research; craft and share nuanced reflections on how data collection methods should be revised or modified; reflect on the epistemological bases of qualitative research; or argue that some qualitative practices should end. Share your thoughts on Twitter using the hashtag: #aqualspace

‛When we shortcut the physical exam, when we lean towards ordering tests instead of talking to and examining the patient, we not only overlook simple diagnoses that can be diagnosed at a treatable, early stage, but we [lose] much more than that. We are losing a ritual. We are losing a ritual that I believe is transformative, transcendent, and is at the heart of the patient-physician relationship’ [1].

‛Health professionals have begun to do research and to write about how doctors and nurses can more effectively listen to their patients. Patient-centred care and relationship-centred care require respectful authentic relationships with patients that come about through attentive and creative listening’ [2].

In this high-tech age, it is easy to overlook the transformative nature of relationship and the power of listening as a route to knowledge. Abraham Verghese reminds us that in diagnosing and treating illness, shortcutting relationship can be fatal; Rita Charon underscores the need for effective listening in building relationships and caring for patients. This clinical wisdom applies as well to the practice of research, where the question of how to listen has been taken up by the Listening Guide [3, 4]. Building on the insights that led to *In a Different Voice *[5], the Listening Guide tunes our ear to the multiplicity of voices that speak within and around us, including voices that speak at the margins and those which in the absence of resonance or response, tend to be held in silence.

In listening for what is unspoken as well as for what is said, for contradiction and for the ways in which one voice can interrupt or silence another, and in recognizing that we often do not say what we mean or say it indirectly, the Listening Guide is a psychological method. It is attentive to the logic of psychological processes and also to the social and cultural frameworks that affect what can and cannot be spoken or heard. The Guide resembles other qualitative methods in incorporating aspects of thematic and narrative analysis as well as elements of a grounded theory approach, but it differs in specifying a series of ‛listenings,’ including the innovative Listening for the ‛I’ (the first person voice of the speaker) and Listening for Contrapuntal Voices (the counterpoint of voices that speak to the researcher’s question). In introducing the Guide, we will illustrate these listenings with a case example drawn from a pilot study with adult daughters of mentally unstable mothers.

But first, a crucial distinction. Reflecting on the training of educators and mental health professionals, the psychiatrist Jonathan Shay, notable for his work with Vietnam combat veterans, observes that ‛all too often our mode of listening deteriorates into intellectual sorting, with the professional grabbing the veteran’s words from the air and sticking them into mental bins.’ Shay thus differentiates the act of listening from what is commonly referred to as coding (sorting what someone says into categories). Although he recognizes the usefulness and the need for categories, he also sees their limitation, noting that ‘at its worst our educational system produces counsellors, psychiatrists, psychologists and therapists who resemble museum-goers whose whole experience consists in mentally saying: *That’s Cubist! That’s El Greco!* and who never *see* anything they’ve looked at’ [6]. It takes a certain discipline or restraint to listen without immediately categorizing or coding what one is taking in, yet doing so opens a space for surprise and thus for discovery.

## Asking a real question

All research starts with a question. There is a difference, however, between questions where the intention is to diagnose or assess others according to a set of criteria or scale, and questions where others are approached not as subjects for assessment but as experts on their own experience. In coming from a place of genuine curiosity or not knowing, the researcher becomes open not only to surprise or discovery but also to having one’s view of the world shaken. When Shay advises that ‛before analyzing, before classifying, before thinking, before trying to *do *anything – we should *listen,*’ he is recounting his experience of having his assumptions about post-traumatic stress disorder unsettled by listening to the stories of veterans. It is also true that this way of listening creates trust and engages others in the research because the act of listening in itself is a manifestation of respect.

Notice what happens when you replace judgment with curiosity. Given how readily we move to judgment, it can be helpful in accessing one’s curiosity to start with the research question and ask: Where does this question come from? Where is it rooted in my experience (my life) and in my field (or in what field of questions does it arise)? Then, given the temptation to prove what we know, it is useful to draw a line down a page and on one side write everything you know about your question, and only when this list has been exhausted, write on the other side of the line what you don’t know and want to know. In this way, your inquiry becomes focused on discovering what you don’t know.

In the illustration that follows, the real question is: how do adult daughters with difficult mothers navigate this relationship? In this instance, replacing judgment with curiosity meant putting aside assessing whether it was better for daughters to stay in relationship with mentally unstable mothers or to break off contact. Instead, the question became what could be learned about staying in or leaving problematic relationships by listening to daughters of difficult mothers? The daughters then were the informants – people with expertise in navigating difficult relationships – rather than subjects of psychological assessment.

## The Listening Guide

The act of listening is not straightforward. What is said directly may differ from what is implied. People can say seemingly contradictory things, like children can both love and hate their parents, and everything said is not of equal weight or value. Qualitative analysis thus presents different challenges from quantitative analysis, and the Listening Guide takes up these challenges by specifying a series of listenings:

Four questions about voice and relationships set the parameters of the inquiry:

- Who is speaking and to whom?
- In what body or physical space?
- Telling what stories about which relationships?
- In what societal and cultural frameworks?

In our example, taken from an interview conducted by Jessica, the person speaking is Penny (a pseudonym), an adult daughter of a mentally unstable mother. Penny is speaking to Jessica, a friend and in this instance also a researcher who approached Penny as an expert on navigating difficult relationships. Penny and Jessica are both adult white women with similar social class and cultural background, and they meet twice over a six-month period, each time in Penny’s apartment for approximately two hours.

The stories Penny tells centre on her relationship with her mother, starting when Penny was a child, spanning her adolescence, continuing into her adult life, and into imagining the future. As a 27-year-old, middle class, white woman raised in the suburbs of New York City, Penny speaks at an intersection of age, class, race, and gender within East Coast American culture.

Given a history of abandonment and the pain she continues to feel when attempting to engage with her mother, Penny questions whether to stay in relationship with her. Approaching Penny as an expert in negotiating a psychologically difficult predicament – what to do in a seemingly irreparable yet irreplaceable relationship – Jessica asks: how does she navigate such a complicated terrain? Penny replies:

‛I don’t know. I won’t let her; I won’t let her be motherly and I won’t – because I don’t like it and I can’t accept it. But I – if you spend enough time with someone on a surface level you can’t help – I have caught myself, [long pause], acting like a daughter. And not acting, like pretending, but doing something that I’m not consciously going: *don’t do that, don’t do that with her.* She was here once for Christmas, and I played a song on the ukulele for her, because she asked me to. And she loved it. And I instantly, in retrospect, I couldn’t handle it. I went to the bathroom and started crying. I couldn’t, I was like, *oh no,* like, *oh no, no, no, no, what did I just do?* I just opened a door of wanting her to say that I was good, or wanting her to be a parent and say *oh, you’re really great,* like, *oh you have such a nice voice,* like, *that’s so great.* It was like, [sigh], an out of body experience.’

## Three listenings

The Listening Guide specifies three successive ‛listenings,’ each guiding a different path through the narrative. The first, Listening for the Plot, surveys the terrain, asking what are the features that distinguish this particular psychological landscape: who is present, is anyone missing, what are the major and minor themes, are there emotional hotspots, salient images or metaphors, what stories are told, are there gaps or ruptures in the narrative, and also what is the researcher’s response to being on this landscape with this person?

In the excerpt from Jessica’s interview with Penny, we find ourselves on a terrain of conflict. Penny ‛won’t let’ her mother ‛be motherly’ and yet she catches herself ‛acting like a daughter.’ We note the words and phrases she uses to describe her predicament – ‛caught myself,’ ‛acting,’ ‛pretending,’ ‛consciously,’ ‛played,’ ‛opening a door,’ ‛wanting,’ and ‛out of body experience.’ An emotional hotspot is when in response to her mother’s request, she plays a song on the ukulele and ends up crying in the bathroom. She speaks of wanting her mother to be a parent as ‛opening a door’ and also as ‛an out of body experience.’ The gaps between her conscious intention and what she can’t help doing, between wanting and then not being able to handle getting what she wants, along with the phrase ‛I don’t know’ as a prelude to saying what in fact she knows, mark this as a landscape of dissociation.

With this first listening, our aim is to be descriptive, to be specific, to stay close to what she says and to use her words wherever possible. If at this stage we analyze or interpret what we are hearing, the likelihood is that we are assimilating what we have taken in to what we already know. In this instance, we might be curious about Penny’s references to acting or pretending, moved by her inability to handle the feelings aroused by her mother’s loving her playing, and we might ask what was it about the time with her mother at Christmas that led Penny to have ‛an out of body experience?’ Jessica also would reflect on how listening to Penny affected her, in order to differentiate her thoughts and feelings, including about mother daughter relationships, from Penny’s.

The second listening is called Listening for the ‛I’ and here one attends to the first-person voice of the other, asking how the ‛I’ speaks of acting and being on this particular psychological landscape. Separating each I phrase (subject and verb) from the narrative and listing it in the order of its appearance, one composes an ‛I poem,’ with each ‛I’ starting a new line of the poem and stanza breaks indicating where the I shifts direction or where a singer might pause for breath. For example, in the above excerpt from the interview with Penny, the I poem begins:

I don’t knowI won’t let herI won’t let herI won’tI don’tI can’tII’m not.

In composing an I poem, there are two rules: (1) highlight every I phrase within a given passage, (2) record these phrases in the order of their appearance in the passage. As the above illustration shows, one can include the object where it seems appropriate to do so (‛I won’t let her’).

In another part of the interview, we hear the I moving from knowing to not knowing and then giving voice to desire:

I knowI haveAm I ever?I don’t knowI don’t haveI don’t haveI want

Continuing with this poem, we hear the I questioning, thinking, asking, not knowing, and finally being her (mother).

Am I going?Am I gonna?I thinkI’ll be leftAm I really ok?Am I really ok?I canI don’t knowI meanI’m her

I poems lay bare the associative logic of a particular psyche as it crosses a specific terrain. In this example, we hear Penny’s I struggle between knowing and not knowing, between having and not having, a possible hidden desire, and confusion between herself and her mother. Following the associative logic, we recognize patterns in the way this I moves, from positive assertions: ‛I know/I have’ – to negations: ‛I don’t know/I don’t have.’ In this way, we may be tapping into unconscious processes.

Attending to a first-person voice, we hear the many ways in which an individual speaks of themselves. In contrast to most qualitative methodologies, the Listening Guide takes into account and addresses the mind’s ability to dissociate or push knowledge and experience out of conscious awareness. In particular, listening for the I may shed light on dissociated knowledge expressed in the first-person voice (‛I don’t know’), and like a poem illuminate the ways in which our minds work in deep connection to our emotions. Listening for the I thus highlights an associative logic, rather than linear, rational, causal thought processes [7].

The third and final listening is called Listening for Contrapuntal Voices. Also a unique step and an innovation specific to this method, Listening for Contrapuntal Voices attends to the participant’s voice not for its content or themes but for its quality or musicality. This means listening for different voices and their interplay, or harmonies or dissonances within the psyche, tensions with parts of itself. This step not only picks up on what is being said, and being said differently at different times, but it is also sensitive to what is not being said or what may be silenced. Listening for different voices and their counterpoint further nuances our understanding of the data by resisting binary categories or dichotomies. Critical in this last step is to use one’s research question as a touchstone and to listen for and identify voices that inform the inquiry. Without the question to guide us, we can readily become lost in a cacophony of orchestral sound, as we are all capable of speaking in a myriad of ways and in a number of different voices.

To illustrate this step, two voices that sound distinct within Penny’s interview (among four that were defined and tracked) were an Angry voice and a Vulnerable voice (named by the researcher). Penny’s Angry voice sounds raw and honest, unpolished, unfiltered, rapid, and loud. Speaking in lists and using profanity, the Angry voice abruptly interrupts other voices mid-sentence. In contrast, Penny’s Vulnerable voice can almost be heard blushing, speaking softly, slowly, and at times muffled or trailing off into pauses and nervous laughter. In the following quote, Penny is speaking to the possibility of forgiving her mother. Penny’s Angry voice is represented by text in italics, while her Vulnerable voice is noted with bold lettering:

‛I’m still like actively defending my dad. **That like – he did right by me [laughs] and you – contin – continue to be even in like [deep breath] the best – or you’re being so nice, like –** *you’re still shit. You’ve always been shit, in addition to just being shit, I almost wish you would’ve been absent. You were actively horrific. Like, you would show up just to fuck shit up. And like, to just take that out of my personality and to take it out of like my whole entire life’s experience just* – **[pause] it – I, I don’t know,** I don’t know what I’m left with if I just go, *ohhkay.*’

This quote shows how Penny’s Angry voice interacts with her Vulnerable voice: the Angry voice taking control, cutting-off, and covering what the Vulnerable voice might say if it had the chance to speak longer.

After the researcher has completed these three listenings – Listening for the Plot, Listening for the I, and Listening for Contrapuntal Voices (with multiple listenings often occurring within each step) – the challenge is to assemble the evidence gained through these listenings and on the basis of this evidence, to compose an analysis. Returning to the research question, the real question, we have found it helpful to ask: what was surprising? Was there a ‛wow’ moment in the interview or within the process of the listenings? If so, what was it and why did it wow you? Using the evidence gathered through the guided listenings, the researcher now brings their voice back as the composer of the analysis, showing clearly the lines that led from evidence to interpretation. This also makes it possible for others to follow the path taken by the researcher, and to question any given interpretation by showing how the evidence might be heard or understood differently.

## Applications of the Listening Guide

The Listening Guide has been used across a wide range of contexts and projects including studies of depression, eating disorders, sexual decision making, adolescent boys’ friendships [8–12] as well as research with women serving in combat units in the Israeli army, transcripts of trials of male witches in early modern Germany, and mothers of former child soldiers in Uganda [13–15]. It is applicable whenever there is a first-person voice, including in the analysis of diaries, letters, speeches, narratives, Supreme Court opinions using the first person, and so forth. Developed and refined over a period of several decades, it has an impressive track record for discovery [16–32]. In addition to its use in analyzing narratives or interview data, the Listening Guide has been adapted for use with video recordings of group discussions and family therapy sessions [33, 34].

## Concluding reflections

Later in the interview with Penny, when she reflects on her ability to shut her mother out, she says, ‛it’s as easy as not picking up the phone. I could just let her go to voicemail all the time – like not a problem.’ The researcher could have remained silent and taken this statement as fact. In doing so, she would likely have concluded that ignoring her mother was easy for Penny, ‛not a problem.’ Instead, by listening closely and picking up on the word ‛could,’ she hears the conditional and responds by saying, ‛but you don’t do that, do you?’ To which Penny replies in a voice that sounds less defensive and exhausted, ‛no, I don’t do that and I hate that.’

This is what effective listening and transformative relationship entail. When Penny says ‛I could just let her go to voicemail,’ Jessica grasps that she is telling her that in fact she doesn’t do this. By asking Penny is this true, she then comes to a deeper understanding of the predicament. The Listening Guide method thus asks the researcher in the interest of discovery to be fully attentive and present in the moment, to listen closely and actively respond, to engage rather than disengage. In this way, it challenges us to rethink what we mean by objectivity.

The Listening Guide is both a method and a methodology, a way of working with a distinctive logic or epistemology. It reframes the research process as a process of relationship, guiding both data collection and data analysis. Seen in this light, authentic relationship and responsive listening become integral to the process of discovery.
